# Exploring the practical themes for medical education social accountability in Iran

**Published:** 2015

**Authors:** Soleiman Ahmady, Maryam Akbari Lakeh

**Affiliations:** *Department of Medical Education, School of Medical Education, Shahid Beheshti University of Medical Sciences, Tehran, Iran*

**Keywords:** Socially accountable medical education, Practical themes, Expert panel discussion

## Abstract

**Aim**: The purpose of this paper is to explore themes for enhancing socially accountability in medical education.

**Background**: Medical education in Iran experience new challenges due to the enormous influence of changes in technology, development of new methods of teaching and learning, student requirements, patient management, financial credit constraints, and social and economic developments. For responding to these, use of strategic thinking in order to make appropriate decisions is the only solution. Strategic plans need to formulate practical guides which can help accountable to people's reasonable expectations.

**Patients and methods**: For this qualitative study, along with the 14^th^national conference on Medical Education in Iran, the opinions of experts were obtained during seven expert panels’ group discussions, each lasting four hours and including 10 participants. Data were collected by audiotapes, which were then transcribed. Data analyzed using a thematic content analysis approach. Peer and member checking during analysis and data triangulation from other recent studies were used to increase the findings’ trustworthiness.

**Results**: Among more than hundred meaning units groups identified the following eight main themes as affecting the social accountable medical education in Iran: organization of responsive education councils; development of community based courses; development in field training; organization of educational processes; homogeneity in educational rules and regulations; budget management, educational outcomes; educational programs in departments and groups.

**Conclusion**: This study have found the main themes that might affecting social accountable medical education in Iran, where Iranian policymakers should consider those when plan to make changes in medical education and could potentially adopt the proven useful policies and strategies of other countries.

## Introduction

Accountability and social commitment is one of the philosophical approaches to higher education, which is focused on responsibility of universities to serve the community. This approach rooted in higher education potentials of solving problems and social issues. Socially accountable medical education trained doctors who are able to serve their community and effectively encounter with the problems (1, 2). 

Accountability is one of the main components in health system management and policy making. Accountability in health system means: “outcomes and benefits for customers which are fulfilled when, inner and outer organizational relationship designed with sufficient knowledge of and appropriate response to the customers' reasonable expectations. In fact, accountability meets reasonable expectations of the individuals to the non-medical aspects of the health system. Reasonable expectations are recognized and accepted as the principles and standards or rules (3). 

Legal accountability means the extent to which officials comply with laws and regulations or are accountable to judicial authorities. Moral accountability is based on the belief that compliance and adherence to moral and spiritual values must be formed within the ​​in education, which needs suitable training (4, 5). Political accountability limits the abuse of political power (6). For the functional and structural accountability in an organization, in addition to establishing the proper foundation for achieving the ambitious goals, needs to know how to use organizational resources and its short-term and immediate effects on organizational performance (7). Cultural accountability means any organization is governed by specific behaviors. There are specific values to the affairs in the world, which can be observed in the behaviors, thoughts, goals, structure, rules, policies, goals, job descriptions of the organization. In this view, organizational culture is a miniature of the general culture (8). 

Today medical practitioners are faced with different challenges than the previous doctors. These challenges include: the changing social, economic, and demographic. As a result of raising the patients’ expectations, and the emphasis on primary care, community oriented medical education (COME) and community based education (CBE) has become more important (9). COME is out of the third or second level health services which have been established in hospitals; here, the community should be included in the educational program (10, 11). CBE is based on learning the dealing with illness and disease through the realities of community. For social accountability, Policy makers and program planners should be aware of the community health system, facilities and learning conditions, and know the community transition conditions with coordination of educational programs with community need changes (13). For responding to these challenges, the use of strategic thinking in order to make appropriate decisions is the only solution. Strategic plans need to formulate practical guides which can help accountable to people's reasonable expectations. The purpose of this paper is to explore practical themes for enhancing social accountability in medical education. 

## Material and Methods

Using qualitative research by applying content thematic analysis methodology along with the 14^th^ conference on medical education in Iran, the opinions of experts were obtained during seven expert panels’ group discussions, each lasting four hours and including 10 participants. The participants were chosen from among who have had experience with community based services and effective administrations of educational programs were done by purposive sampling. We wanted to bring together a diverse group in order to maximize our exploration of different perspectives within a group setting. The ultimate goal of these meetings was to analyze the main themes of social accountable medical education in the context of Iran. Therefore, the participants were invited through Minister of Health and Medical Education (MOHME), and the importance of the sessions was emphasized for them.

As we wished to focus on the factors perceived as influencing the social accountable medical education, all the experts at sessions emphasized this topic. Each session included two moderators: one who was familiar with medicine and medical education, and one who was familiar with management and foresight planning. During the first session, we explained the study concept and clarified the goals of the meetings, and then asked questions regarding potential world factors that could affect Iran's medical education system. 

Next, participants were asked to discus and list what they perceived as being accountable medical education. The conversations were recorded on audiotapes and transcribed after the session. Brainstorming was allowed to continue until no new opinions were forthcoming. 

We performed an interim analysis because we intend to use the results in the other sessions. Two researchers independently read the transcripts and discussion notes, and then analyzed the data using a thematic framework approach. Based on this analysis, a list was prepared for the other sessions.

In the sessions, participants were asked to review and prioritize the themes list, and then think about the impacts these themes could have on medical education, and also consider possible strategies to deal with these impacts. We concentrated on the impacts and strategies in continuous sessions, using data-gathering and analytic methods similar to those used for the first session. A final result was prepared by combining the results from all panel sessions.

Simultaneous to these panels’ discussion groups, a literature review on themes affecting social accountable medical education and potential ameliorating strategies that have been used in Iran and other countries was performed. We used this review to confirm the validity of our results and compare Iranian strategies with those used in other countries. In an effort to increase the credibility of our results, we used data triangulation and member check (14). 

## Results

With more than hundred meaning units the panel experts agreed on 8 themes that they felt had the largest effects on the accountability in health system and medical education in Iran (table 1).

## Discussion

The themes explored in panel discussions were approximately consistent with those found which in other studies of factors influencing accountability in medical education. Some proposed themes in those studies are discussed below. For example, a philosophical base for developing Community Education programs is provided through the five components of the Wisconsin Model of Community Education. The model provides a process framework for local school districts to implement or strengthen community education (15); the Wisconsin model includes the following 9 themes: 

“1) Self-determination: local people are the best ones to identify their community needs and demand. 

2) Self-help: people do their best when they participate in community programs.

3) Leadership development: use of the leadership capacities of local people are prerequisites for self-help and community programs improvement efforts. 

4) Localization: community opportunities and equipment should bring closest to where people live; this has the greatest effect on more public participation. 

5) Integrated Delivery of Services: Organizations with the same purposes can establish close working relationships with each other for maximum efficacy.

6) Maximum Use of Resources: maximum responding to the diverse needs and interests of the community, it is better to interconnect any kind of resources.

7) Inclusiveness: for full development of the community programs, inhibit any isolation of people in community.

8) Responsiveness: Community has a responsibility to develop programs that respond to the continually changing needs of their constituents.

9) Lifelong Learning: learning opportunities should be available to all ages in a wide variety of community settings (15)”. Therefore, evidences call Iranian medical education system to undergo changes. It seems that, fostering community responsive research; strategic thinking and critical problem solving is necessary for medical education. If so, community people should getting more participate and encourage facilitating their own educations, increasing their motivation, encouraging community based education and life-long learning (16). In coming years, medical education in Iran will need to undergo major changes for accountability. When planning for these changes, decision-makers should consider the various factors that affect social accountable education. Train students who can enhance healthcare system and increase the international rank of Iranian medical universities; should be our ultimate purpose of medical education. Examining the relevant themes over different time courses should allow us to forecast the future of a community’s health and educational needs. Factors affecting social accountability in medical education should be considered in our policies and strategies as they were used by other countries to promote accountability. Also an examination of the policies and strategies used by other countries may facilitate policy planning for the future of medical education in Iran, given our finding that many issues in medical education are comparable worldwide.

**Table 1:** 8 themes that panel experts agreed as the largest effects on the accountability in health system and medical education in Iran

Main Themes	Themes description
(1):Organization of responsive education councils	This theme was identified by many of the participants. For example, one said, “*I am sure that with establishment of responsive educational councils, COME and CBE formed in Iran. This will correspondingly effect on learning activities.*” Incrementally proper management through these councils, helps solving problems in the field of management responsive education. Equitable distribution of funds and Interaction with insurances, are the other benefits of these councils.
(2):Development of community based courses	One participant said: “*When I was student, the education was mostly subject based. These days, I see more holistic approach. Therefore, future attention should be given to the community*.” setting up courses based on the needs of the community, design and implementation of training courses in climate cone, developing new technologies based on the needs of the community, preparation for the unconventional events are the subthemes in this section.
(3):Development in field training	In recent years, rapid growth in medical education has supported the evolution of new fields. Create a standard teaching hospital, establish mentor strengthening of general hospitals, enable clinical teaching centers and educational laboratories are the essentials for field training development.
(4):Organization of educational processes	The education process is expected to undergo many changes in the future, including the increased use of innovative educational techniques, the recognition of relevant community needs and etc. Other aspects that could greatly affect accountability in medical education are potential changes in the analysis of educational processes, shorten the process, perform quality monitoring, establishment of clinical governance, use of Virtual Learning in the theoretical training.
(5):Homogeneity in educational rules and regulations	To social justice and eliminating discrimination in society, the same rules and regulations of the education for all is another theme that groups proposed for social accountability. *“In recent years, we see increasing attention being paid to equity in education, especially in deprived areas. We must take this into account more when planning for medical education.”* said a group member who participated in our discussions. Therefore, it could be useful to place more emphasis on rules and regulations within the medical education system.
(6):Budget management	During our discussions, one expert said, “*We must address the problem of budget when setting policies, especially for education, because it is one major reason for deficit is the social accountable education. It is needed for better educational opportunities and situations*.” Equitable distribution of funds in light of the priorities, monitoring the productivity indicators, remove procedural costs, need additional research on budget management based on each context and should be used to develop strategies aimed at keeping accountability in the country.
(7):Educational outcomes	Training graduates who are competent, caring, committed and aware of the community needs; use strategies for achieving educational excellence; equitable graduates distribution and supply human forces in deprived areas; performance evaluation of graduates based on community needs; transforming knowledge into technology; research directed towards solving people's problems; improve health services indicators; monitoring indicators of professional ethics; collaboration with the family physician; development of traditional medicine; culture for traditional medicine; and active participation of people in education are the most proposed outcomes that should better be considered in social accountable education.
(8):Educational programs in departments and groups	The experts agreed on the following items for development of COME and CBE in departments and groups including: periodical need assessment in each region and announced them to upstream; the use of inter-sect oral experts in training programs development committees; awareness training programs for teachers and students; awareness of program standards; provide facilities; standard monitoring parameters; monitoring progress related constraints; continuous monitoring of training workshops to develop faculty; announced student admission capacity; monitoring patients and their interactions and promotion associated indicators and programs to meet the people rights; employee participation in decision making; education for human dignity; departments engagement in educational activities; setting up multidisciplinary groups and teams in education, research, treatment and prevention.
